# Sequence and Haplotype Analyses of *Ligula intestinalis* in *Acanthobrama marmid* (Cyprinidae) in Turkey

**DOI:** 10.1007/s11686-023-00762-2

**Published:** 2024-01-08

**Authors:** Harun Kaya Kesik, Figen Celik, Cebrahil Turk, Seyma Gunyakti Kilinc, Sami Simsek, Abdurrahman Gul

**Affiliations:** 1 Department of Parasitology, Faculty of Veterinary Medicine, University of Bingol, 12000 Bingol, Turkey; 2 Department of Parasitology, Faculty of Veterinary Medicine, University of Firat, 23119 Elazig, Turkey; 3 Department of Fisheries, Genç Vocational School, Bingol University, 12500 Bingol, Turkey

**Keywords:** *Ligula intestinalis*, Ligulosis, Fish, Haplotype, Turkey

## Abstract

**Purpose:**

Ligulosis caused by *Ligula intestinalis* adversely affects the fisheries carried out in the lakes and ponds, causing economic losses in the fish industry. In this study, it was aimed to reveal the molecular characterization of *L. intestinalis* isolates obtained from woodfish (*Acanthobrama marmid*) in Keban Dam Lake in Elazig province of Turkey by using mt-CO1 gene sequences and to determine the genetic differences and haplotypes between the isolates.

**Methods:**

In the examination made in terms of *L. intestinalis*, the intestine of the fish was opened with the help of fine-tipped scissors, the contents were allowed to come out, and the parasites were taken into a petri dish containing phosphate buffered saline (PBS). Then, *L. intestinalis* plerocercoids were taken into 15 ml falcon tubes containing 70% ethanol and stored at − 20 °C until further analysis. From each isolate, total gDNA was extracted from the plerocercoids. A partial (480 bp) mt-CO1 gene was amplified by PCR and sequenced unidirectionally. The final size of the trimmed sequences was 392 bp for 43 sequences. Sequence and haplotype analyses were performed, followed by phylogenetic analyses.

**Results:**

All isolates were confirmed as *L. intestinalis* by BLAST analysis. In addition, 87 nucleotide mutation positions were determined among 43 CO1 gene sequences. As a result of the haplotype network performed for the mt-CO1 gene region of *L. intestinalis* isolates; arranged in a star-like configuration with the main haplotype (Hap05), separated from other haplotypes by 1–6 mutation steps, and 29 haplotypes were identified, covering 13.9% (6/43) of the total isolates. Also, 75 variable (polymorphic) sites were determined, 52 of which were parsimony informative sites.

**Conclusions:**

The molecular characterization of *L. intestinalis* in woodfish (*A. marmid*) was identified for the first time in Turkey.

## Introduction

Seafood is a cheap and rich source of protein to cover animal protein needs, and it is an industry branch that represent a significant proportion of the world's nutritional needs [1]. Fish infection,, especially parasites, causes significant problems in aquatic ecosystems. Sometimes even a parasite species can threaten the existing reserves [2]. *Ligula intestinalis* (Linnaeus, 1758) ranks first as cestodes among the parasitic agents of fish. It has a significant prevalence in Turkey and creates a problem especially in dam lakes [3]. Ligulosis caused by *Ligula intestinalis* (Linnaeus, 1758) adversely affects the fisheries carried out in the lakes and ponds of in Turkey [4, 5].

*Ligula intestinalis* (Linnaeus, 1758), classified in the *Diphyllobothriidae* family, is a cestode reaching 28 cm as adults and 40 cm as plerocercoids [6, 7]. It is common in fresh waters in the Northern Hemisphere, including Turkey. *Ligula intestinalis;* has been reported in many fish families such as *Cyprinidae, Catostomidae, Salmonidae or Galaxiidae.* However, it shows a remarkable heterogeneity in host preference according to the geographical area studied [8]. Nowadays, the species in the *Ligula* taxon are classified as two genus (*Ligula* and *Digramma*) and four species *(Ligula intestinalis* (Linnaeus, 1758)*, Ligula colymbid* (Zeder, 1803) *and Digramma interrupta* (Rudolphi, 1810)*, Digramma nemachili* (Dubinina, 1957)) according to their morphological and anatomical features [9]. *Ligula intestinalis* is a pseudophyllidean cestode species scolex typically has dorsal and ventral bothriums. Sometimes bothriums are absent, and instead they have slits in the central part of the dorsal and ventral sides. The neck is quite short, almost absent. There are procercoid and plerocercoid stages in their development [10–12].

*Ligula intestinalis* has a three-host life cycle. There are two larval stages, procercoid and plerocercoid. The dominant phase of it life cycle is the plerocercoid phase, which lives in the body cavity of fish [10–12]. The first intermediate hosts are some copepod species. The larval form in these intermediate hosts are procercoids. The second intermediate hosts are freshwater fish, and their larval form is plerocercoids. Waterfowl such as seagulls, ducks, water divers and sea divers are the final hosts [13]. Freshwater fish become infected by eating parasitic copepods [3, 13, 14].

Plerocercoids of *L. intestinalis* most commonly develop in tench, red-eye, perch and pike perch. These plerocercoids are found in the abdominal cavity of fish. It causes swollen and the swimming slows down. In severe infections, the abdomen bursts and causes the death of the fish. Plerocercoids exert mechanical pressure on the reproductive organs and destroy the gonads. However, it is known that it adversely affects reproductive physiology by secreting antigonadotropic hormone. This effect causes infertility and pernicious anemia in fish [15]. In the fight against ligulosis, it is not practical and almost impossible to fight with copepod species belonging to the *Cyclops* and *Diaptomus* genus, which are the first intermediate host. In order to reduce the number of adult parasite-carrying and fish-eating waterfowl, measures should be taken periodically around the lake. Infected fish that move passively in the water should be collected and destroyed as much as possible [16–18].

Molecular techniques are used to identify species that cannot be distinguished morphologically according to their genetic differences [19]. Nuclear 28S rDNA and mitochondrial cytochrome c oxidase subunit 1 (mt-CO1) gene sequencing of *L. intestinalis* are used for molecular identification of the parasite [20]. In the case that both the existing fish species in aquatic ecosystems and the species that are grown intensively in net cages are exposed to this parasitic effect, molecular identification is important avoid economic depreciation and to take the necessary measures [18].

In this study, it was aimed to reveal the molecular characterization of *L. intestinalis* obtained from woodfish (*Acanthobrama marmid*) in Keban Dam Lake in Elazig province of Turkey by using mt-CO1 gene sequences and to determine the genetic differences and haplotypes between the isolates.

## Materials and Methods

### Study Area and Sampling

The fish to be examined for the study were obtained from Keban Dam Lake (Coordinates: 38°50′39″N 39°14′24″E) in Elazig province of Turkey between June and August, 2021. The fish were caught by fishing with gill nets number 22 in the lake. The fish, either alive or dead, were procured from the fishermen and brought to the Bingol University Faculty of Veterinary Fisheries Diseases Laboratory with ice in styrofoam boxes. After the external examination of the fish, the internal examination was carried out in accordance with the necropsy technique and examined for *L. intestinalis* plerocercoids. In the examination of *L. intestinalis*, the intestine of the fish was opened with the help of fine-tipped scissors, the contents were allowed to come out, and the parasites were taken into a petri dish containing physiological water. Then, *L. intestinalis* plerocercoids were taken into 15 ml falcon tubes containing 70% ethanol and stored at -20 °C until further analysis. The terminal segments were stained with aceto-carmine and mounted with Canada balsam. The specimens were identified as *L. intestinalis* by using taxonomic keys [21]. The morphological characterization of plerocercoid was completed by observing them under a light microscope.

### Genomic DNA Isolation

Total genomic DNA (gDNA) was extracted using a Hibrigen Genomic DNA Isolation Kit (Hibrigen, Kocaeli-Turkey) according to the manufacturer’s instructions, with some slight modifications. The samples were washed five times using phosphate buffered saline (PBS) to remove the alcohol and then the plerocercoids was cut into small pieces with a scalpel, and transferred into Eppendorf tubes (1.5 ml). Two hundred microliters of lysis solution and 20 µl of Proteinase-K (20 mg/ml) were included in the tubes, mixed, then incubated overnight at 65 °C. To support the lysis, the samples were vortexed every 1 h for at least two to three times. The next day kit procedures were followed and total gDNA was isolated. The quantity of gDNA was (Nanodrop Technologies, Wilmington) measured and those samples containing insufficient gDNA were re-extracted to obtain a sufficient amount of gDNA. Isolated samples were stored at − 20 °C until further use.

### Amplification of mt-CO1 Gene Fragment by PCR

To determine the genetic diversity of *L. intestinalis* isolates, the mt-CO1 gene region (480 bp) was amplified with the specific primers COIA2 (5’CATATGTTTTGATTTTTTGG3’) and COIB2 (5’AKAACATAATGAAAATGAGC3’) as previously described [22]. The PCR reaction comprised: 10 X PCR buffer (5 µl), MgCl_2_ (25 mM), dNTP’s (400 uM), from each primers (20 pmol), TaqDNA Polymerase (5 U/µl), PCR distilled water (28.8 µl) and gDNA (5 µl). The thermal cycling parameters of the PCR reaction were as follows: pre-denaturation for 5 min at 95 °C; 35 cycles of 45 s at 95 °C, 45 s at 57 °C, and 1 min at 72 °C, and extra extension step for 5 min at 72 °C. A Blue-Ray Biotech from (Taiwan, R.O.C) thermal cycler was used for PCR amplification and PCR products were separated using 1.4% agarose gel during 30 min. at 90 V constant current.

### DNA Sequences

The PCR products of the mt-CO1 gene belonging to *L. intestinalis* isolates (*n* = 50) were sent to a commercial company (DNA Laboratory Systems, Istanbul, Turkey) for one-way Sanger DNA sequence analyses.

### Data Analysis of the Sequences

FinchTV 1.4.0 was utilized to analyze the sequence data. BLAST search (www.ncbi.nlm.nih.gov/BLAST/) has been used to compare and trim our nucleotide sequences with published reference sequences. The MEGA X software was used to evaluate the trimmed sequences [23]. Using NCBI PubMed published reference sequences as outgroups, alignment of the sequences was performed. *Ligula intestinalis* (MZ359928, NC039445) was used as the reference sequence for phylogenetic tree construction while *Taenia saginata* (AY684274) and *Echinococcus granulosus* (NC044548) sequences were used as outgroup. The most suitable phylogenetic tree model for the sequences were identified as Tamura-Nei + Gamma distribution (+ G) by the MEGAX program. The phylogenetic tree was constructed using the Maximum Likelihood method in MEGA X, after finding the most accurate and suitable DNA model [23].

### Nucleotide Diversity, Polymorphism and Neutrality Indices and Haplotype Networks of the Sequences

All sequence data were uploaded into DNASP6 software for more comprehensive evaluation as described by Rozas et al. [24]. The software was utilized to identify the diversity of neutrality and population indices. Population diversity indices included nucleotide diversity (πd), haplotype diversity (hd) and haplotype number (hn), while neutrality indices consisted of Tajima’s D [25], Fu’s statistics [26], Fu’s Fs [26], Fu and Li’s D test (FLD), Fu and Li's F (FLF) statistics test and parsimony informative analysis were performed using DnaSP6. Data in a number of output formats, such as NEXUS, was created using the DNASP6 (DNA Sequence Polymorphism) software program. To generate the haplotype network, the Minimum Spanning Networks (MSN) method was used in the PopART-1.7 software (Population Analysis with Reticulate Trees) [27, 28]. Haplotype analysis of the 43 sequences with a size of 392 bp belonging to the mt-CO1 gene fragment of *L. intestinalis* isolates was performed.

## Results

**Fig. 1:**
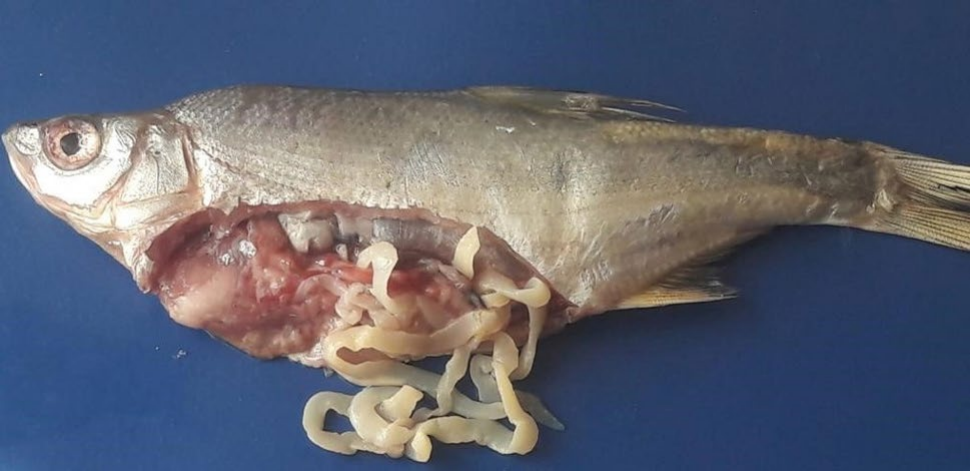
Plerocercoids of *L. intestinalis* spilling out of the body cavity of a *Acanthobrama marmid*

A total of 50 *L. intestinalis* plerocercoids were obtained from the abdominal cavity of fishes (*A. marmid*) (Fig. 1). To identify the exact species of the isolates, the mt-CO1 gene fragment (480 bp) was amplified using PCR. After sequence analysis, nucleotide sequences were compared with published reference sequences. The final size of the trimmed sequences was 392 bp and 7 sequences were excluded because of the low quality sequence reads although twice attempts. Totally 43 sequences of mt-CO1 samples were submitted to GenBank.

**Fig. 2:**
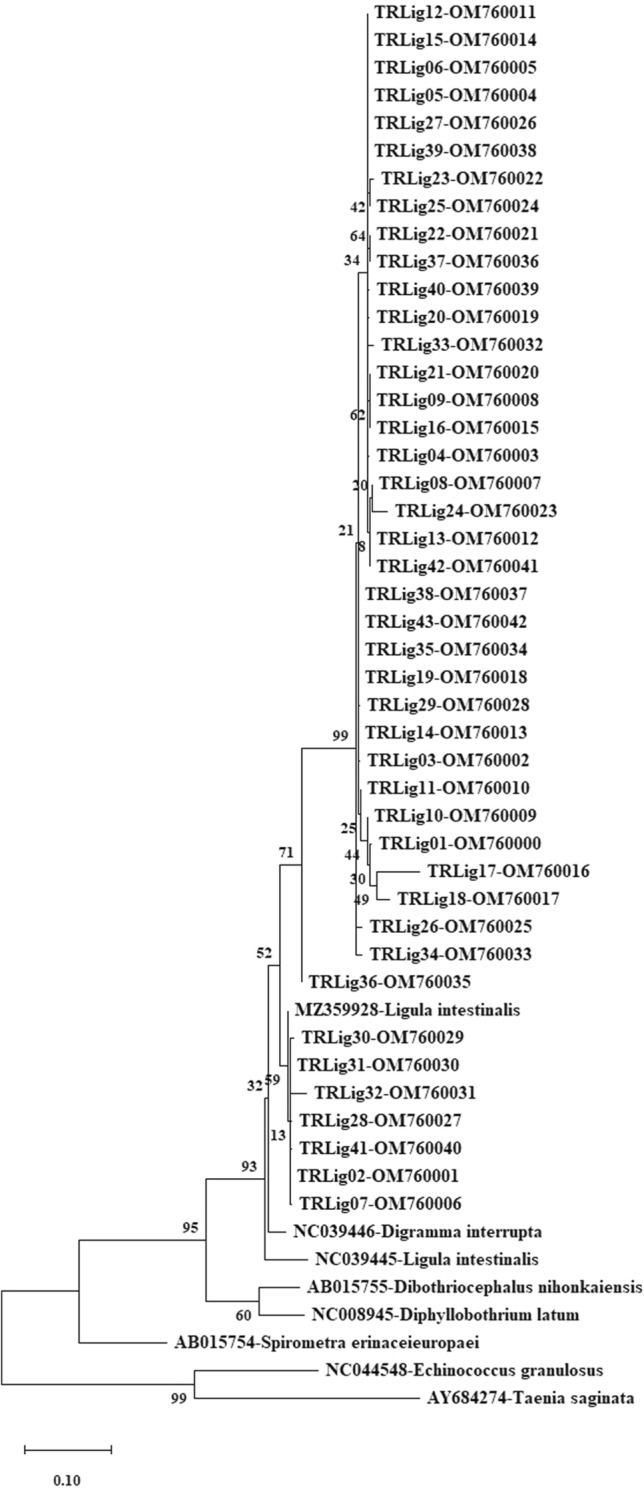
Phylogenetic tree view created using reference sequences from *L. intestinalis* (MZ359928, NC039445) and outgroup sequence *Taenia saginata* (AY684274), *Echinococcus granulosus* (NC044548) with 392 bp *mt*-*CO1* gene sequences (TRLig001-TRLig43) of *L. intestinalis* isolates. The tree was created based on the Tamura-Nei + Gamma distribution (+ G) (TN93 + G) model with the Maximum Likelihood method in MEGA-X, and the reliability of the tree was ensured by 1,000 bootstrap tests

Following the alignment, point mutations in the sequences were detected. Nucleotide variation positions based on the reference sequence are demonstrated in the Table 1. A phylogenetic tree was generated with the bootstrap test (1000 replicates) by applying the Maximum Likelihood statistical method and is shown in Fig. 2.

**Table 1:** Nucleotide variation positions of the mt-CO1 (392 bp) gene among 29 haplotypes analysed

Nucleotide positions	14	18	21	22	25	27	31	34	35	36	39	40	42	45	46	48	54	59	60	63	69	72	78	92
NC_039445 (Reference sequence)	G	T	C	T	A	G	C	G	G	T	T	T	T	T	G	C	T	T	C	C	A	A	A	T
Hap01		C	T				T			C				C		T			T	T	G			
Hap02			T				T			C	C					T					G		G	
Hap03		C	T				T			C				C		T			T	T	G			
Hap04		C	T				T			C				C		T			T	T	G			
Hap05		C	T				T			C				C		T			T	T	G			
Hap06			T				T			C	C				C	T					G		G	
Hap07		C	T				T			C				C		T			T	T	G			
Hap08		C	T				T			C	C			C		T			T	T	G			
Hap09		C	T				T			C				C		T			T	T	G			
Hap10		C	T				T			C				C		T			T	T	G			
Hap11		C	T				T			C				C		T			T	T	G			
Hap12		C	T				T			C				C		T			T	T	G			
Hap13		C	T				T	T	C	C				C		T			T	T	G			
Hap14	T		T	C	T		T			C				C		T			T	T	G			
Hap15		C	T				T			C				C		T			T	T	G			
Hap16		C	T				T			C				C		T			T	T	G	G		
Hap17		C	T				T	C		C				C		T		A	T	T	G			
Hap18		C	T				T	T		C		G	G	C		T			T	T	G			
Hap19		C	T				T			C				C		T		A	T	T	G			
Hap20		C	T				T			C				C		T		A	T	T	G			
Hap21			T				T			C	C					T					G		G	
Hap22		C	T				T			C				C		T			T	T	G			
Hap23			T				T			C	C					T					G		G	
Hap24			T				T			C	C					T	C			T	G		G	C
Hap25	T		T	C			T			C				C		T			T	T	G			
Hap26		C	T			T	T			C				C		T			T	T	G			
Hap27		C	T				T			C				C		T					G			
Hap28		C	T				T	T		C				C		T			T	T	G			
Hap29			T				T			C	C					T					G		G	

Nucleotide positions	93	96	99	102	105	108	117	120	123	132	136	138	142	145	150	157	159	162	165	167	171	172	174	180
NC_039445 (Reference sequence)	C	T	A	T	A	C	T	C	C	A	G	T	G	G	G	G	T	T	C	G	A	G	C	G
Hap01			G		G	T	A	T	T	G			T						T	T			T	T
Hap02	T				G	T	C										C	C	T				T	
Hap03					G	T	A	T	T	G									T				T	
Hap04			G		G		A	T	T	T									T				T	
Hap05			G		G		A	T	T										T				T	
Hap06	T				G	T	C										C	C	T				T	
Hap07			G		G		A	T	T	T									T				T	
Hap08			G		G	T	A	T	T										T				T	
Hap09			G		G	T	A	T	T	G									T	T			T	T
Hap10			G		G		A	T	T	G									T				T	
Hap11			G		G	T	A	T	T										T				T	
Hap12			G		G		A	T	T	G									T				T	
Hap13			G	C	G	T	A	T	T	T		A	T	T	T	T			T	T	C	T	T	T
Hap14			G		G		A		T	T		A	T						T	T	C		T	T
Hap15			G	C	G		A	T	T										T				T	
Hap16			G		G		A	T	T										T				T	
Hap17			G		G		A	T	T										T				T	
Hap18			G		G		A	T	T	T	A								T				T	
Hap19			G		G		A	T	T										T				T	
Hap20			G		G	T	A		T	G		A							T				T	
Hap21	T				G	T	C										C	C	T				T	
Hap22			G		G	T	A	T	T	G									T				T	
Hap23	T				G	T	A										C	C	T				T	
Hap24	T	C		C	G	T	C		T	G							C	C	T				T	
Hap25			G		G		A	T	T										T				T	
Hap26			G		G	T	A		T	G									T				T	
Hap27			G		G	T	A										C	C	T				T	
Hap28			G		G		A	T	T										T				T	
Hap29	T				G	T	C										C	C	T				T	

Nucleotide positions	182	186	188	189	193	195	196	198	199	201	202	204	213	222	225	231	240	243	253	255	273	282	285	288
NC_039445 (Reference sequence)	T	T	G	C	C	A	A	T	G	C	A	A	T	G	C	T	C	T	T	A	A	C	T	C
Hap01								A		A			A	T	T	C			C	G	G	T	C	T
Hap02				T						T							T	C	C					T
Hap03								A		A				T	T	C			C	G	G	T	C	T
Hap04								A		A				T	T	C			C	G	G	T	C	T
Hap05								A		A				T	T	C			C	G	G	T	C	T
Hap06				T						T							T	C	C					T
Hap07								A		A			A	T	T	C			C	G	G	T	C	T
Hap08								A		A				T	T	C			C	G	G	T	C	T
Hap09						T		A		A			A	T	T	C			C	G	G	T	C	T
Hap10								A		A			A	T	T	C			C	G	G	T	C	T
Hap11								A		A			A	T	T	C			C	G	G	T	C	T
Hap12								A		A				T	T	C			C	G	G	T	C	T
Hap13	A		C		A	T	C		T	A		T	A	T	T	C			C	G	G	T	C	T
Hap14								A		A			A	T	T	C			C	G	G	T	C	T
Hap15								A		A				T	T	C			C	G	G	T	C	T
Hap16								A		A				T	T	C			C	G	G	T	C	T
Hap17								A		A			A	T	T	C			C	G	G	T	C	T
Hap18						T		A		A	C		A	T	T	C			C	G	G	T	C	T
Hap19								A		A				T	T	C			C	G	G	T	C	T
Hap20								A	T	A				T	T	C			C	G	G	T	C	T
Hap21				T						T							T	C	C			T		T
Hap22								A		A				T	T	C			C	G	G	T	C	T
Hap23				T						T							T	C	C					T
Hap24				T													T	C	C					T
Hap25								A		A				T	T	C			C	G	G	T	C	T
Hap26		C						A		A				T	T	C			C	G	G	T	C	T
Hap27										A						C		C	C			T		T
Hap28								A		A				T	T	C			C	G	G	T	C	T
Hap29				T				C		T							T	C	C					T

Nucleotide positions	294	297	301	306	318	321	399	342	345	350	351	366	369	373	381
NC_039445 (Reference sequence)	T	T	A	G	C	T	G	A	T	T	T	A	T	G	C
Hap01	C			A	T	C		G			A	G			T
Hap02	A	C			T		A	G	C				C		T
Hap03	C			A	T	C		G			A	G			T
Hap04	C		T	A		C		G			A	G			T
Hap05	C		T	A		C		G			A	G			T
Hap06	A	C			T		A	G	C				C		T
Hap07	C		T	A		C		G			A	G			T
Hap08	C		T	A		C		G			A	G			T
Hap09	C			A	T	C		G			A	G			T
Hap10	C			A	T	C		G			A	G			T
Hap11	C		T	A		C		G			A	G			T
Hap12	C			A	T	C		G			A	G			T
Hap13	C		T	A		C		G			A	G			T
Hap14	C			A	T	C		G			A	G			T
Hap15	C		T	A		C		G			A	G			T
Hap16	C		T	A		C		G			A	G			T
Hap17	C		T	A		C		G			A	G			T
Hap18	C		T	A		C		G			A	G		A	T
Hap19	C		T	A		C		G			A	G			T
Hap20	C			A	T	C		G			A	G			T
Hap21	A	C			T		A	G	C				C		T
Hap22	C			A	T	C		G		A	A	G			T
Hap23	A	C			T		A	G	C		A		C		T
Hap24	A	C			T		A	G	C				C		T
Hap25	C		T	A		C		G			A	G			T
Hap26	C			A		C		G			A	G			T
Hap27	A	C			T		A	G	C				C		T
Hap28	C		T	A		C		G			A	G			T
Hap29	A	C			T		A	G	C				C		T

**Fig. 3:**
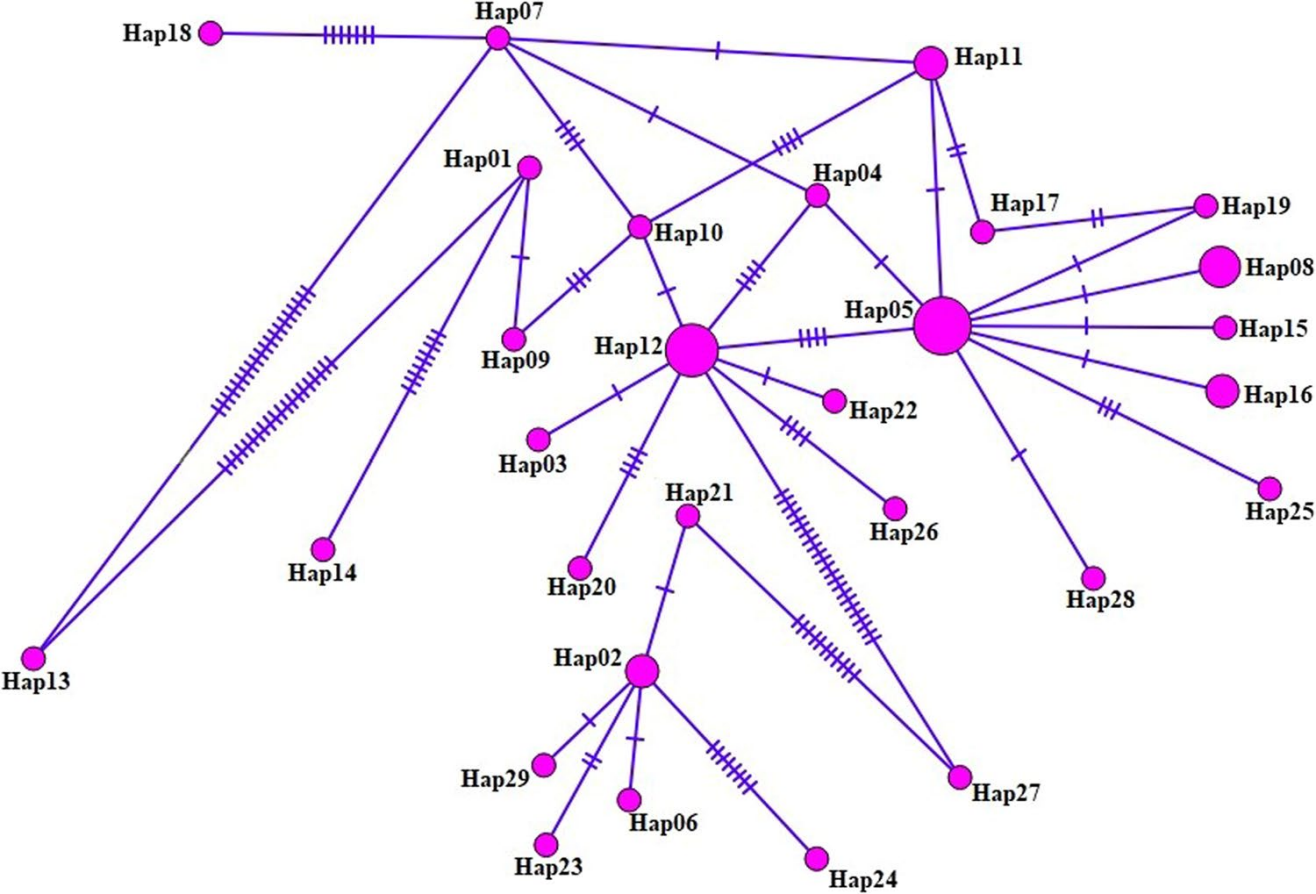
The haplotype network for the *mt-CO1* (392 bp) gene of *L. intestinalis*. The size of the circles is proportional to the frequency of each haplotype. The number of mutations separating haplotypes is indicated by dash marks. Hap Haplotype

As a result of the haplotype network performed for the mt-CO1 gene region of *L. intestinalis* isolates; arranged in a star-like configuration with the main haplotype (Hap05), separated from other haplotypes by 1–6 mutation steps, and 29 haplotypes were detected, covering 13.9% (6/43) of the total isolates. (Fig. 3; Table 2). As a result of haplotype analysis, 75 variable (polymorphic) sites were determined, 52 of which were parsimony informative sites. Low nucleotide diversity and high haplotype variation were reported for the mt-CO1 gene fragment (Table 3). Tajima’s D value was demonstrated population expansion and/or refinement of selection. Uncommon haplotypes from recent population expansion or hitchhiking were expected because of the significant negative Fu’s Fs value. Therefore, the fact that 79.3% (23/29) of the haplotype groups were single haplotypes supported these results. The sequences identity among the published reference sequence (NC_039445) and the current sequences (OM760000-OM760042) for *L. intestinalis* are presented in Table 2. After a BLAST search, several novel *L. intestinalis* haplotypes arose. The Hap02 and Hap29 were matched 100% with the published sequences in the Genbank. Although, Hap06 and Hap21 had 99.74% similarity rate, Hap13 had the lowest similarity with 90.67%. Finally, 27 haplotypes were determined as unique haplotypes in the current study (Table 4).

**Table 2:** Haplotypes of mt-CO1 sequences of *L. intestinalis* and accession numbers of isolates forming groups

No	Haplotype name	Number of isolates	Isolate codes (Accession numbers)
1	Hap01	1	TRLig01 (OM760000)
2	Hap02	2	TRLig02 (OM760001) TRLig31 (OM760030)
3	Hap03	1	TRLig03 (OM760002)
4	Hap04	1	TRLig04 (OM760003)
5	Hap05	6	TRLig05 (OM760004) TRLig06 (OM760005) TRLig12 (OM760011) TRLig15 (OM760014) TRLig27 (OM760026) TRLig39 (OM760038)
6	Hap06	1	TRLig07 (OM760006)
7	Hap07	1	TRLig08 (OM760007)
8	Hap08	3	TRLig09 (OM760008) TRLig16 (OM760015) TRLig21 (OM760020)
9	Hap09	1	TRLig10 (OM760009)
10	Hap10	1	TRLig11 (OM760010)
11	Hap11	2	TRLig13 (OM760012) TRLig42 (OM760041)
12	Hap12	5	TRLig14 (OM760013) TRLig19 (OM760018) TRLig35 (OM760034) TRLig38 (OM760037) TRLig43 (OM760042)
13	Hap13	1	TRLig17 (OM760016)
14	Hap14	1	TRLig18 (OM760017)
15	Hap15	1	TRLig20 (OM760019)
16	Hap16	2	TRLig22 (OM760021) TRLig37 (OM760036)
17	Hap17	1	TRLig23 (OM760022)
18	Hap18	1	TRLig24 (OM760023)
19	Hap19	1	TRLig25 (OM760024)
20	Hap20	1	TRLig26 (OM760025)
21	Hap21	1	TRLig28 (OM760027)
22	Hap22	1	TRLig29 (OM760028)
23	Hap23	1	TRLig30 (OM760029)
24	Hap24	1	TRLig32 (OM760031)
25	Hap25	1	TRLig33 (OM760032)
26	Hap26	1	TRLig34 (OM760033)
27	Hap27	1	TRLig36 (OM760035)
28	Hap28	1	TRLig40 (OM760039)
29	Hap29	1	TRLig41 (OM760040)

**Table 3:** Diversity and neutrality indices obtained using nucleotide data of the *L. intestinalis* *mt*-CO1 gene (392 bp)

*n*	H	Hd+SD	πd ± SD	Tajima’s D	*p* value	Fu’s Fs	*p* value	FLD	*p* value	FLF	*p* value
43	29	0,966±0,016	0,03962±0,00643	− 0,53754	*p* > 0.10	− 5,060	0.004	0,78869	*p* > 0.10	− 0,83226	*p* > 0.10

*n *number of isolates, *H* number of haplotypes, *h*d haplotype diversity, *π*d nucleotide diversity, *SD* standard deviation, *FLD* Fu and Li’s D* test statistic, *FLF* Fu and Li’s F* test statistic

**Table 4:** Sequence identity (%) between mt-CO1 gene lineages of *L. intestinalis*

	NC_039445	Hap01	Hap02	Hap03	Hap04	Hap05	Hap06	Hap07	Hap08	Hap09	Hap10	Hap11	Hap12	Hap13	Hap14	Hap15	Hap16	Hap17	Hap18	Hap19	Hap20	Hap21	Hap22	Hap23	Hap24	Hap25	Hap26	Hap27	Hap28	Hap29
NC_039445	100.00	89.29	92.60	90.82	90.82	91.07	92.35	90.56	90.82	89.54	90.31	90.82	90.56	85.97	88.78	90.82	90.82	90.31	88.78	90.82	90.05	92.35	90.31	92.35	91.07	90.82	90.56	92.60	90.82	92.35
Hap01		100.00	89.80	98.47	97.70	97.70	89.54	97.96	97.45	99.74	98.98	97.96	98.72	94.90	97.70	97.45	97.45	97.45	96.68	97.45	97.70	90.05	98.47	90.31	89.54	96.94	97.70	92.86	97.45	89.80
Hap02			100.00	91.33	90.31	90.56	99.74	90.05	90.82	90.05	90.82	90.31	91.07	85.46	89.29	90.31	90.31	89.80	88.27	90.31	90.56	99.74	90.82	99.49	97.96	90.31	90.56	96.94	90.31	99.74
Hap03				100.00	98.72	98.72	91.07	98.47	98.47	98.72	99.49	98.47	99.74	93.37	96.68	98.47	98.47	97.96	96.68	98.47	98.72	91.58	99.49	91.84	91.07	97.96	98.72	93.88	98.47	91.33
Hap04					100.00	99.74	90.05	99.74	99.49	97.96	98.72	99.49	98.98	94.64	96.43	99.49	99.49	98.98	97.96	99.49	97.96	90.56	98.72	91.84	89.80	98.98	98.47	95.15	99.49	90.31
Hap05						100.00	90.31	99.49	99.74	97.96	98.72	99.74	98.98	94.39	96.17	99.74	99.74	99.23	97.70	99.74	97.96	90.82	98.72	91.07	89.80	99.23	98.47	93.62	99.74	90.56
Hap06							100.00	89.80	90.56	89.80	90.56	90.05	90.82	85.20	89.03	90.05	90.05	89.54	88.01	90.05	90.31	99.49	90.56	99.23	97.70	90.05	90.31	96.68	90.05	99.49
Hap07								100.00	99.23	98.21	98.98	99.74	98.72	94.90	96.68	99.23	99.23	99.23	98.21	99.23	97.70	90.31	98.47	99.23	89.54	98.72	98.21	93.11	99.23	90.05
Hap08									100.00	97.70	98.47	99.49	98.72	94.13	95.92	99.49	99.49	98.98	97.45	99.49	97.70	91.07	98.47	91.33	90.05	98.98	98.21	93.37	99.49	90.82
Hap09										100.00	99.23	98.21	98.98	94.64	97.45	97.70	97.70	97.70	96.94	97.70	97.96	90.31	98.72	90.56	89.80	97.19	97.96	93.11	97.70	90.05
Hap10											100.00	98.98	99.74	93.88	97.19	98.47	98.47	98.47	97.19	98.47	98.72	91.07	99.49	91.33	90.56	97.96	98.72	93.88	98.47	90.82
Hap11												100.00	98.72	94.64	96.43	99.49	99.49	99.49	97.96	99.49	97.70	90.56	98.47	90.82	89.54	98.98	98.21	93.37	99.49	90.31
Hap12													100.00	93.62	96.94	98.72	98.72	98.21	96.94	98.72	98.98	91.33	99.74	91.58	90.82	98.21	98.98	94.13	98.72	91.07
Hap13														100.00	94.13	94.64	94.13	94.39	94.13	94.13	93.62	85.71	93.37	85.97	85.46	93.62	93.11	88.52	94.64	85.20
Hap14															100.00	95.92	95.92	95.92	94.90	95.92	96.94	89.54	96.68	89.80	88.78	96.94	96.43	91.84	95.92	89.29
Hap15																100.00	99.49	98.98	97.45	99.49	97.70	90.56	98.47	90.82	90.05	98.98	98.21	93.37	99.49	90.31
Hap16																	100.00	98.98	97.45	99.49	97.70	90.56	98.47	90.82	89.54	98.98	98.21	93.37	99.49	90.31
Hap17																		100.00	97.70	99.49	97.70	90.05	97.96	90.31	89.03	98.47	97.70	92.86	99.23	89.80
Hap18																			100.00	97.45	95.92	88.52	96.68	88.78	87.76	96.94	96.43	91.33	97.96	88.27
Hap19																				100.00	98.21	90.56	98.47	90.82	89.54	98.98	98.21	93.37	99.49	90.31
Hap20																					100.00	90.82	98.72	91.07	90.31	97.19	98.47	93.62	97.70	90.56
Hap21																						100.00	91.07	99.23	97.70	90.56	90.82	97.19	90.56	99.49
Hap22																							100.00	91.33	90.56	97.96	98.72	93.88	98.47	90.82
Hap23																								100.00	97.45	90.82	91.07	96.94	90.82	99.23
Hap24																									100.00	89.54	90.31	93.62	89.54	97.70
Hap25																										100.00	97.70	92.86	98.98	90.31
Hap26																											100.00	93.62	98.21	90.56
Hap27																												100.00	93.37	96.68
Hap28																													100.00	90.31
Hap29																														100.00

Sequence identity (%) between the reference genome of *L. intestinalis* and the mt-CO1 gene strains of the haplotypes detected in this study

## Discussion

One of the health problems encountered in fish farming is parasitic diseases. In recent years, it has been reported that parasitic diseases are quite common in freshwater fish farming in our country. The diseases not only have harmful effects such as growth retardation and reproductive problems in fish, but also lead to death when the parasite population is high [29]. The recognition of parasitic fish diseases and the investigation of their treatment are of great importance for the developing fishery sector today. Parasites are encountered intensively when the fish density is high, malnutrition and environmental conditions change. Weakness and intense parasitic invasions developing with increasing stress factors can be fatal for the fish [17, 30, 31].

In recent studies in Turkey, parasitic diseases are frequently encountered in fish species belonging to the *Cyprinidae* family found in natural waters [32–36]. Turkey has a rich location in terms of both its geographical location and water resources (lake, river, sea, etc.) and fish population. Thus, parasitic infections encountered in fish facilities and fisheries are very important for Turkey's economy. In our study, the presence of *L. intestinalis* infection in the *A. marmid* species was investigated and the internal examinations of the fish were examined by necropsy technique and 50 *L. intestinalis* plerocercoids were obtained from the abdominal cavities of the fishes. In this study, detection of *L. intestinalis* parasite in *A. marmid* was in agreement with the studies in terms of detection of the parasite in freshwater fish. In some studies, the changing in *L. intestinalis* prevalence depend on the seasons, age groups and sex characteristics of the fish were investigated [34, 37–39]. Moreover, the effect of the parasite on the fish condition factor and physiological-anatomical structures was also investigated [40–46].

Today, PCR-based DNA sequence analysis and other molecular methods are frequently preferred for the identification of parasite agents and their phylogenetic characterization [8]. Studies on *L. intestinalis* species are mostly anatomical-morphologically based in Turkey [47], and the molecular studies are limited [48]. However, genetic markers such as ITS and CO1 have been frequently used in recent years to accurately determine the taxonomic positions of organisms [20]. The traditional classification of ligulid cestodes *Ligula* (Bloch, 1782) and *Digramma* (Cholodkovsky, 1914) is a controversial issue. Especially, the low nucleotide diversity between the genus *Ligula* and *Digramma* indicates that *Digramma* is probably not an independent genus. For this reason, it showed that *Ligula* and *Digramma* should be considered as two species within the genus *Ligula*. As a result of the studies, it has been reported that ITS1, ITS2 and ND1 sequences are useful genetic markers to distinguish between *Ligula* and *Digramma* [49–52]. Lagrue et al. [52] reported a case of *Ligula sp.* in fish species *Gobiomorphus cotidianus* and *Oncorhynchus tshawytscha* from Lake Hawea, South Island of New Zealand. Parasites have been quite recorded in large sizes (60–300 mm). In addition, low prevalences in fish populations suggested that the infection was rare or local. The ITS1 and ITS2 sequences confirmed that these isolates belong to the *Ligula* genus [14]. In present study, sequence of the mt-CO1 gene fragment was preferred to determine the genetic diversity and phylogenetic characterization of *L. intestinalis* isolates collected from *A. marmid* fish species belonging to the *Cyprinidae* family. As a result all sequences were match with *L. intestinalis* in the current study.

Several studies have been carried out on the molecular identification of *L. intestinalis* infection, which causes serious yield and economic losses in fish facilities. Li et al. [20], certain nucleotides of 393 bp for the CO1 gene of seven *L. intestinalis* samples were directly sequenced from each sample. Interestingly, no nucleotide variation was detected in the sequences of the CO1 gene among the seven *Ligula* samples studied. The authors reported that this homology in sequences may be due to the mutation patterns of the mitochondrial genome and the sequence similarity of CO1 genes of closely related taxon. In another study, a single pattern and reliable band were recorded in all gene sequences of 6 identified *L. intestinalis* isolates. The size of the bands were determined as 480 bp. No nucleotide variation was detected in any of the CO1 gene products of the samples. Only one mutation was detected at nucleotide 225 of one sample [14]. In support of the data of the above researchers, in this study similar *Ligula intestinalis* specific primers were used. A single pattern and a reliable band were recorded in all of the gene sequences of the 43 *L. intestinalis* isolates. The bands were 480 bp in size. All isolates were confirmed as *L. intestinalis* by BLAST analysis. In addition, 87 nucleotide mutation positions were determined among 43 CO1 gene sequences. This suggests that *L. intestinalis* plerocercoids in *A. marmid* fish show higher nucleotide diversity compared to other fish species.

Bouzid et al. [22], a study was conducted to determine the phylogeny and genetic structure analysis of *L. intestinalis* according to geography and fish host preference. The analyzed data consisted of 109 parasites from 13 host fish species in 18 different locations on a macrogeographic scale. The two mitochondrial genes CO1 and cytb were preferred to determine the population genetic structure of *L. intestinalis* on a local and global scale. Besides, the nuclear sequence of the ITS2 gene was used for genetic reconstruction. Different evolutionary diversity was determined at different locations. Although the ITS2 sequences were found to show significant intragenomic variability, their associations were generally stated to be in good agreement with the topology derived from mitochondrial genes. In another study, the genetic diversity of *L. intestinalis* was analyzed using ISSR markers. In the study, ten host species from nine different locations and nine ISSR markers were used to analyze the genetic diversity of *L. intestinalis* populations. The 110 loci selected from the ISSR patterns produced revealed high variability among the analyzed samples, with a polymorphism of 100% and a global coefficient of gene differentiation estimated by Nei’s index (GST) of 0.776. Major genetic variation was associated with five different geographic locations (Algeria, Canada, Australia, China and Europe). However, no remarkable genetic variation was detected, even though samples in Europe were obtained from different geographic regions and different hosts. In conclusion, the ISSR approach has been shown to be quick and cheap and provides reliable indicators to evaluate the genetic diversity of *L. intestinalis* [8]. In present study, we determined 29 haplotypes among the 43 *L. intestinalis* isolates. We observed 87 multiple nucleotide mutation positions within 29 haplotypes. By analysis of the sequences, the most common haplotypes were Hap05 and Hap12, respectively. Identification of 29 haplotypes in 43 isolates revealed the important genetic diversity of *L. intestinalis* By haplotype analysis, low nucleotide variation and high haplotype diversity were recorded for the mt-CO1 partial gene sequences of *L. intestinalis*. Tajima’s D value indicated expansion of the population and the influence of purifying selection. The negative value of Fu’s Fs indicated that the presence of the rare haplotypes occurred because of hitchhiking or recent population enlargement. Therefore, it was not surprising that there were 29 haplotypes out of 43 sequences. Besides, 79.3% (23/29) of the haplotype consisted of a single sequence.

## Conclusion

This study was conducted by taking into account that it is necessary the molecular characterization of *L. intestinalis*, which causes serious economic losses in fishing. Molecular characterization studies of *L. intestinalis* are limited in Turkey. As a result of this study, the haplotype analysis of *L. intestinalis* was revealed for the first time. Hence, the molecular characterization of *L. intestinalis* in woodfish (*A. marmid*) was identified for the first time. The data of this study will also contribute to the epidemiology, control and treatment options of *L. intestinalis* infection, which is one of the most serious economic problems of the country's fishing. With this study, will open the way for more original and comprehensive research on molecular identification of parasitic disease agents in all regions of our country in the future.
